# Anti-colorectal cancer effects of anti-p21Ras scFv delivered by the recombinant adenovirus KGHV500 and cytokine-induced killer cells

**DOI:** 10.1186/s12885-018-4989-y

**Published:** 2018-11-12

**Authors:** Fang-Rui Liu, Shuang Bai, Qiang Feng, Xin-Yan Pan, Shu-Ling Song, Hong Fang, Jing Cui, Ju-Lun Yang

**Affiliations:** 1 Faculty of Medicine, Kunming University of Science and Technology, Kunming, Yunnan 650500 China; 2 Department of Pathology, Kunming General Hospital, 212 Daguan Road, Kunming, Yunnan 650032 People’s Republic of China

**Keywords:** Ras, Colorectal cancer, Adenovirus, CIK, scFv

## Abstract

**Background:**

Colorectal cancer (CRC) is the most common type of gastrointestinal cancer. CRC gene therapy mediated by adenovirus holds great promise for the treatment of malignancies. However, intravenous delivery of adenovirus exhibits limited anti-tumor activity in vivo when used alone.

**Methods:**

In this study, the antitumor activity of the recombinant adenovirus KGHV500 was assessed with the MTT, TUNEL, Matrigel invasion and cell migration assays. To enhance the intravenous delivery of KGHV500 in vivo, cytokine-induced killer (CIK) cells were used as a second vector to carry KGHV500. We explored whether CIK cells could carry the recombinant adenovirus KGHV500 containing the anti-p21Ras single chain fragment variable antibody (scFv) gene into tumors and enhance antitumor potency.

**Results:**

Our results showed that KGHV500 exhibited significant antitumor activity in vitro. In the nude mouse SW480 tumor xenograft model, the combination of CIK cells with KGHV500 could induce higher antitumor activity against colorectal cancer in vivo than that induced by either CIK or KGHV500 alone. After seven days of treatment, adenovirus and scFv were detected in tumor tissue but were not detected in normal tissues by immunohistochemistry. Therefore, KGHV500 replicates in tumors and successfully expresses anti-p21Ras scFv in a colorectal cancer xenograft model.

**Conclusions:**

Our study provides a novel strategy for the treatment of colorectal cancer by combining CIK cells with the recombinant adenovirus KGHV500 which carried anti-p21 Ras scFv.

## Background

As the most common cancer malignancy worldwide, CRC is the fourth leading cause of cancer related deaths [1]. Radiotherapy and chemotherapy are a “double-edged sword”, that kills cancer cells, but also damages normal cells. Thus, targeted therapy and gene therapy are necessary improvements for colorectal cancer. As far as targeted drugs, cetuximab [2] and panitumumab [3] target the epidermal growth factor receptor (EGFR) and benefit CRC patients with EGFR overexpression, but they are ineffective in patients without EGFR expression [4, 5]. Therefore, it is necessary to identify new therapeutic targets for CRC.

The Ras gene was the first oncogene to be discovered in human tumors and plays a significant role in the development of many tumor types [6]. K-Ras mutations occur in approximately 30–50% of CRC cases [7], and p21Ras is overexpressed in CRC [8, 9].

Our previous studies revealed a high expression rate of wild-type p21Ras in CRC but no expression in normal colorectal epithelia, which together with other data, suggest that p21Ras is an important intracellular target for cancer therapy. However, to date, no drug targeting p21Ras has been approved for clinical use.

In recent years, we prepared anti-p21Ras scFv which could react with mutant p21Ras and wild-type p21Ras proteins [10]. Further study demonstrated that a recombinant adenovirus carrying the gene for anti-p21Ras scFv could penetrate tumor cells, express anti-p21Ras scFv intracellularly and inhibit the proliferation of tumor cells with p21Ras overexpression. Intratumoral injection of the recombinant adenovirus showed intracellular expression of anti-p21Ras scFv and obvious inhibition of transplanted tumor growth. For gene therapy, the SSAT gene [11] and E2F-1 gene [12] carried by adenovirus exhibit significant antitumor activity against CRC in vitro. However, intravenous delivery of adenovirus is still a main problem in gene therapy. To improve the safety of systemic anti-p21Ras scFv delivery for therapy of metastatic and late stage cancers, in this study, we employed CIK cells as a second vector to carry the recombinant adenovirus KGHV500 that harbored the anti-p21Ras scFv gene to tumor foci, and then investigated its anti-colorectal cancer effects.

## Methods

### Cell lines

The human colorectal cancer (CRC) cell line SW480 harbors a K-ras mutation at codon 12 [13] and overexpresses c-Myc [14], and the human embryonic kidney (HEK) 293 cell line was purchased from the Conservation Genetics CAS Kunming Cell Bank (Kunming, CN). CD46 expression on SW480 cells was confirmed by immunohistochemistry (IHC).

HEK293 cells and SW480 cells were grown in the 1640 medium supplemented with 10% heat-inactivated fetal bovine serum (FBS) (Biological Industries, Israel,#64–001-1ACS) under atmospheric conditions of 5% CO_2_ at 37 °C.

### Recombinant adenovirus

Recombinant adenovirus KGHV400 was constructed previously by us based on a wild-type adenovirus (Ad5). In KGHV400 the E1A and E1B promoters were replaced and controlled by the hTERT and HRE promoters. The Ad5 cilia gene was replaced with the Ad35 cilia gene. KGHV500 was constructed by inserting the anti-p21Ras scFv gene into KGHV400. Both KGHV400 and KGHV500 were purified by discontinuous density gradient centrifugation with cesium chloride, and the titers of the recombinant adenovirus was determined by tissue culture infective dose (TCID50) in HEK 293 cells [15, 16].

### Recombinant adenovirus infected tumor cells

The SW480 cells were cocultured with KGHV500 for 48 h and then centrifuged for 5 mins at 800 rpm. Electron microscopy and immunohistochemistry were used to check for KGHV500 infection in SW480 cells. Briefly, the cell pellets were fixed in 3.5% glutaraldehyde for 5–6 h at 4 °C, dehydrated through a graded series of alcohols and acetones and embedded in Epon 618 resin. Then, the tissue blocks were cut into 0.5–2 μm ultrathin sections using an ultrathin microtome (Leica R, Leica company, Germany) and then stained with uranyl acetate. KGHV500 infection was checked with an electron microscope (JEM-1011, Japan). During immunohistochemical staining, a monoclonal antibody recognizing the adenovirus hexon protein was used to detect hexon protein, which was expressed by KGHV500 in SW480 cells.

### Cell migration assay

SW480 cells were splited into 6 well plates and cultured with RPMI 1640 medium containing FBS at a density of 1 × 10^5^ per well. When SW480 cells grew to approximately 80% confluency, a scratch approximately 0.6 mm wide was created with a pipette tip. The image of cell migration over the scratch was captured at 0 h, 24 h and 48 h with a 10× objective lens.

### Cell invasion assay

Transwell chambers (Corning 3422 LOT#12211005) were coated with 60 μl diluted-Matrigel and placed into the incubator for 3 h. The untreated SW480 cells and SW480 cells infected with KGHV500 (3 × 10^4^–1 × 10^5^ cells/ml) were seeded into the Transwell chambers. After incubation, the Transwell membrane was fixed with methanol for 15 min and stained with Giemsa (Solarbio, USA, #G8220) for 15 min. Photographs were taken with an inverted microscope to count the fixed cells (each sample counted 10 fields).

### MTT assay

SW480 cells (1 × 10^4^ cells per well in 96-well plate) infected with the adenovirus at different time points and cell viability evaluated, then, 20 μl MTT(5 mg/ml, PH = 7.4; Amresco, USA) was added. After incubation, DMSO was added and the MTT results were read at 490 nm.

### TUNEL analysis

SW480 apoptosis was detected by TUNEL assay according to the protocol (In Situ Cell Death Detection Kit POD; Roche Diagnostics GMbH, Germany), and the apoptotic SW480 cells exhibited characteristic nuclear fragmentation with green staining [17].

### Preparation and identification of CIK cells

Aliquots of 100 ml human peripheral blood were used to isolate CIK cells as described previously by Loretta et al. [15, 16]. Peripheral blood mononuclear cells (PBMCs) were isolated and cultured at a cell density of 1× 10^6^ cells/ml in RPMI 1640 medium (HyClone, USA,#SH30809.01), with timed additions of 1000 U/ml IFN-γ (PeproTech, USA,#AF-300-02), 50 ng/ml anti-CD3 antibody (PeproTech, USA,#60181–1-Ig) and 300 U/ml recombinant human interleukin IL-2 (PeproTech, USA,#AF-200-02) after 24 h (supplemented with 300 U/ml recombinant human interleukin IL-2 every 3 days until the end of the expansion). The final cell concentrations were adjusted to 2 × 10^5^ cells/ml. CIK cells were identified by immunohistochemistry with anti-CD3 and anti-CD56 antibodies.

### Recombinant adenovirus infected CIK cells

CIK cells were plated in 24-well plates (2 × 10^5^ cells/200 μl cells), cocultured with KGHV500 at an MOI of 100, centrifuged at 900 g for 1 h, and then cultured under atmospheric conditions of 5% CO_2_ at 37 °C for 1 h with 300 μl 10% FBS-supplemented in RPMI 1640 medium. The infection efficiency of the virus infecting CIK cells was detected by immunohistochemistry.

### Establishment of nude xenograft tumors model and tumor measurement

Specific pathogen free (SPF) female BALB/c nude mice at six weeks of age were purchased from Vital River Laboratories (Beijing, China). A total of 1 × 10^7^ SW480 cells were injected by subcutaneous injection into the right subaxillary. 50 mice bearing SW480 tumor xenografts were assigned randomly to five groups when the tumor size reached an average diameter of 5.0 mm. Then, the CIK cells harboring recombinant adenovirus were injected into the tumor-bearing mice by tail vein injection. The injections were repeated a total of 5 times on an every other day injection schedule. Tumor sizes were measured every three days. When the tumor reached 2000 mm^3^ or ulcerated, the mice were sacrificed [18].

### Quantitative real-time RT-PCR for apoptotic gene detection

Total RNA was extracted from tumor tissues using an ReliaPrep™ RNA Cell Miniprep kit (Promega, Madison, WI, USA) according to the protocol. The PCR amplification products were read with Bio-Rad CFX96 Manager software.

### Immunohistochemistry detected adenovirus hexon and scFv

The tumor tissue sections were incubated with 70 μl 10% BSA at 37 °C for 40 min to block nonspecific binding sites, then incubated at 4 °C with anti-Flag Tag Antibody (Cell Signaling Technology, #2368) overnight, and then incubated with secondary antibody for 40 min at 37 °C. A a fresh amount of DAB chromogenic agent (50 μl DAB concentrate in 1 ml DAB substrate solution) was added at room temperature for 5–10 min.

### Western blot analysis

The expression of scFv in the organs of the nude mice was detected by Western blot. Briefly, the nude tissue was ground in a mortar, and lysed in RIPA buffer with PMSF for 30 min to extract all tissue proteins. The protein solution was run on an SDS–PAGE and transferred to polyvinylidene fluoride (PVDF). The PVDF membrane was incubated at room temperature with the anti-Flag Tag Antibody (Cell Signaling Technology, #2368) at 1:1000, and then with secondary antibody at a 1:1000 dilution. β-actin (ZSGB-BIO, Beijing, China,#TA-09) was used as an internal control.

### Statistical analysis

Each statistical analysis was performed using SPSS Version 22.0. Date are expressed as the mean value ± s.d. Statistical significance was performed with one-way ANOVA and the Student–Newman–Keuls method. Statistical significance different was indicated by a value of *P*<0.05.

## Results

### SW480 cell infection by recombinant adenovirus KGHV500

**Fig. 1:**
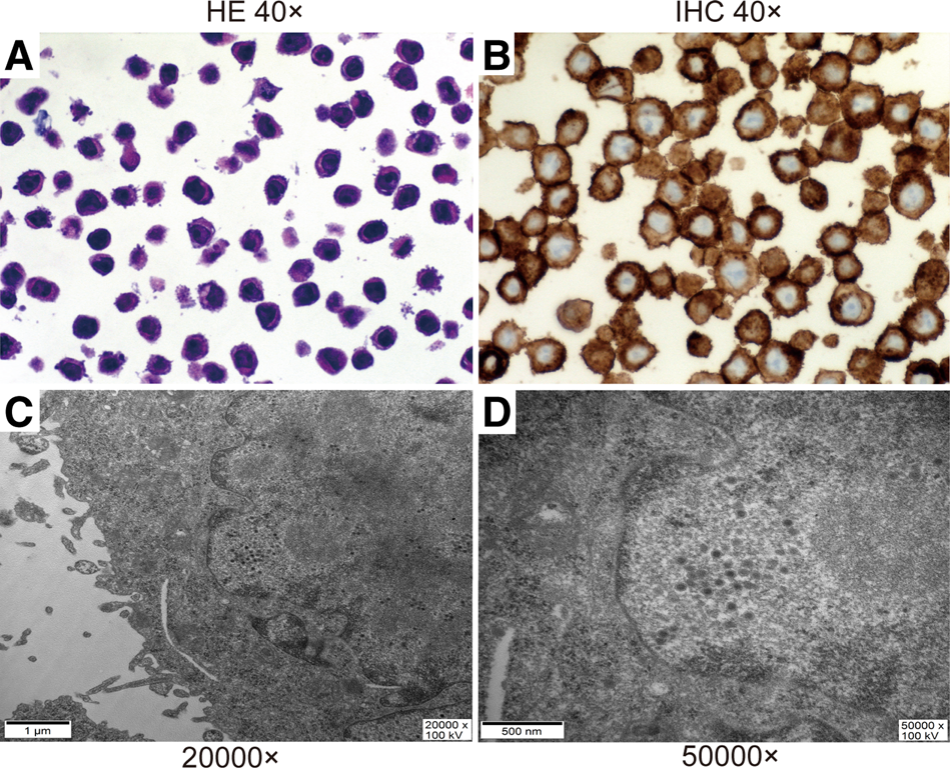
The identification of KGHV500 infected SW480 cells. (**a**) The HE staining of SW480 cells (40×). (**b**) The expression of CD46 on the surface of SW480 tumor cells is high and detected by immunohistochemistry staining. CD46’s and the main location is on the cell membrane (40×). (**c** and **d**) Many virus particles with a diameter of 70–90 nm in the cytoplasm and nucleus of SW480 cells (20000×) and (50000×) were detected by electron microscopy

Since the CD46, receptor of the fiber node of KGHV500 was expressed on the cell membrane of SW480 cells (Fig. 1), KGHV500 could bind to SW480 cells. After coculture with KGHV500, we found many virus particles with of 70–90 nm in the cytoplasm and nucleus of SW480 cells by electron microscopy (Fig. 1c and d), which confirmed that SW480 cells were successfully infected wth KGHV500.

### The anti-tumor efficacy of KGHV500 in vitro

**Fig. 2:**
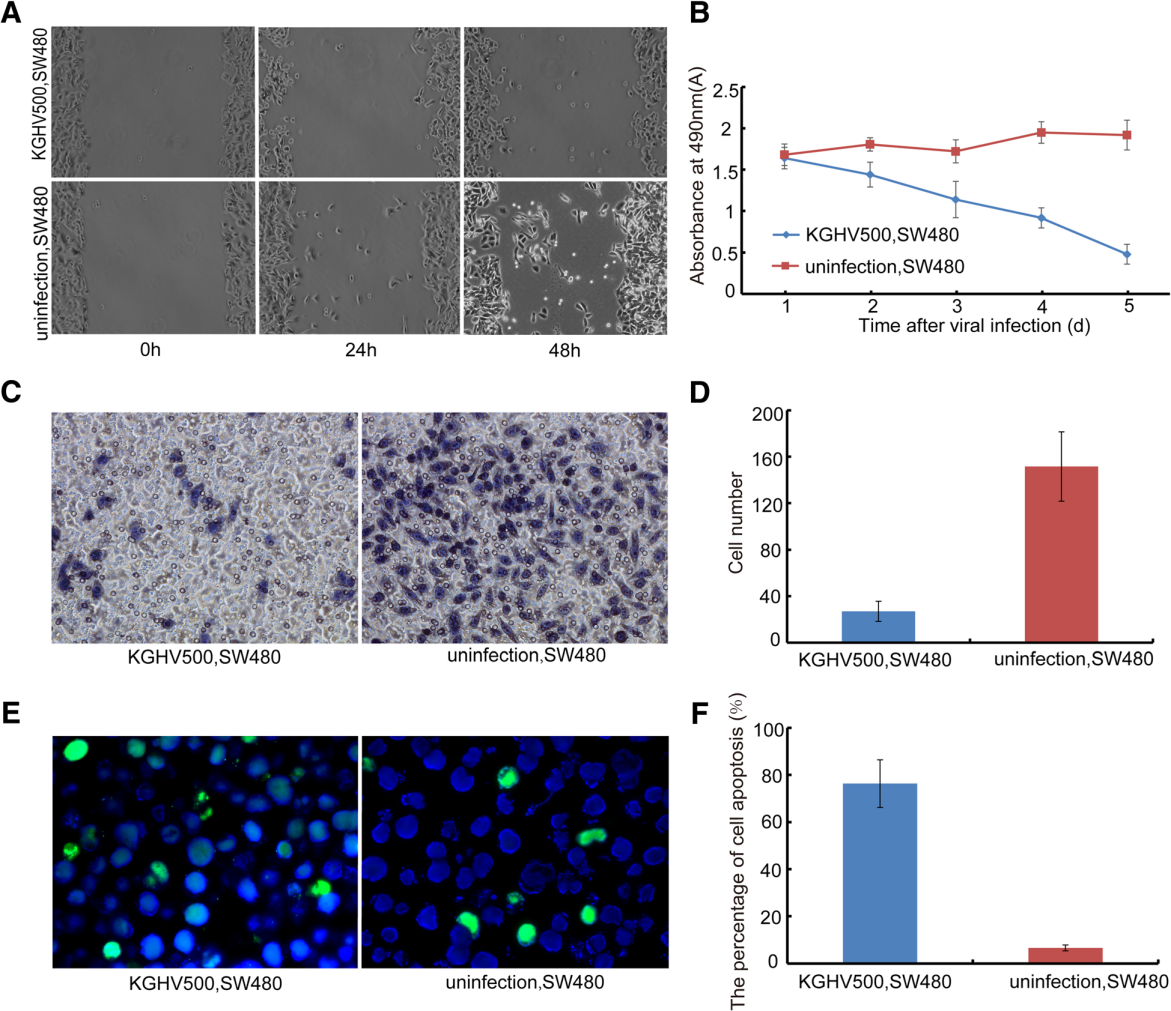
The anti-tumor efficacy of KGHV500 in vitro. (**a**) The result of the cell migration assay using SW480 cells infected by KGHV500 at an MOI of 100.0 for 0 h, 24 h, 48 h. The migration of SW480 cells was inhibited by KGHV500. (**b**) MTT assay of cell growth in SW480 cells treated with KGHV500 for 1d, 2d, 3d, 4d and 5d. The growth of SW480 cells was inhibited by KGHV500. (**c** and **d**) Transwell assay: The number of invading cells in the KGHV500-infected SW480 cells group was much lower than the number of invading SW480 cells in the control group (*P* < 0.01). (**e**) There were more apoptotic tumor cells found in the KGHV500 group by TUNEL staining (green: TUNEL, blue: DAPI). (**f**) The percentage of apoptotic cells was higher in the KGHV500 infected SW480 cells group than in the SW480 control group (*P* < 0.01). Data are presented as the mean ± s.d. *Significantly different from the control group (*P* < 0.05). **P < 0.01 vs controls

A scratch test was performed on SW480 cells that were infected with KGHV500 at an MOI of 100 for 24 h, and the healing of the scratches was observed (Fig. 2a). The percentages of cell scratches were 1.89 ± 1.41% at 24 h and 8.81 ± 4.04% at 48 h after infection. The percentages of cell scratches were 10.52 ± 1.40% at 24 h and 22.58 ± 4.21% at 48 h in the control group (no infection) (*P* < 0.05). These results suggested that KGHV500 could effectively inhibit the migration ability of SW480 cells.

The killing effect of KGHV500 was detected with MTT assay. KGHV500 infection killed half of the SW480 cells, and the absorbance decreased significantly in SW480 cells after 4 d of infection. The results showed that the growth of SW480 cells was inhibited by KGHV500 (Fig. 2b).

A Transwell test was used to detect inhibition of SW480 cell invasiveness by KGHV500. We found that the number of cells that invaded the bottom of the Transwell chamber was 27 ± 8.73 in the KGHV500 infected SW480 cells group, while the number of cells was 151.57 ± 29.90 in the normal control group (*P* < 0.01) (Fig. 2c and d).

The apoptosis of SW480 cells was examined by TUNEL assay. The number of apoptotic SW480 cells increased obviously after KGHV500 infection, and there were almost no apoptotic cells in the uninfected SW480 cells group. The percentage of apoptosis was significantly higher in the infected group 76.34 ± 10.05% than in the control group 6.68 ± 1.25% (*P* < 0.01). The TUNEL results indicated that KGHV500 could significantly promote the apoptosis of SW480 cells (Fig. 2e and f).

### CIK cells bind to the recombinant adenovirus

**Fig. 3:**
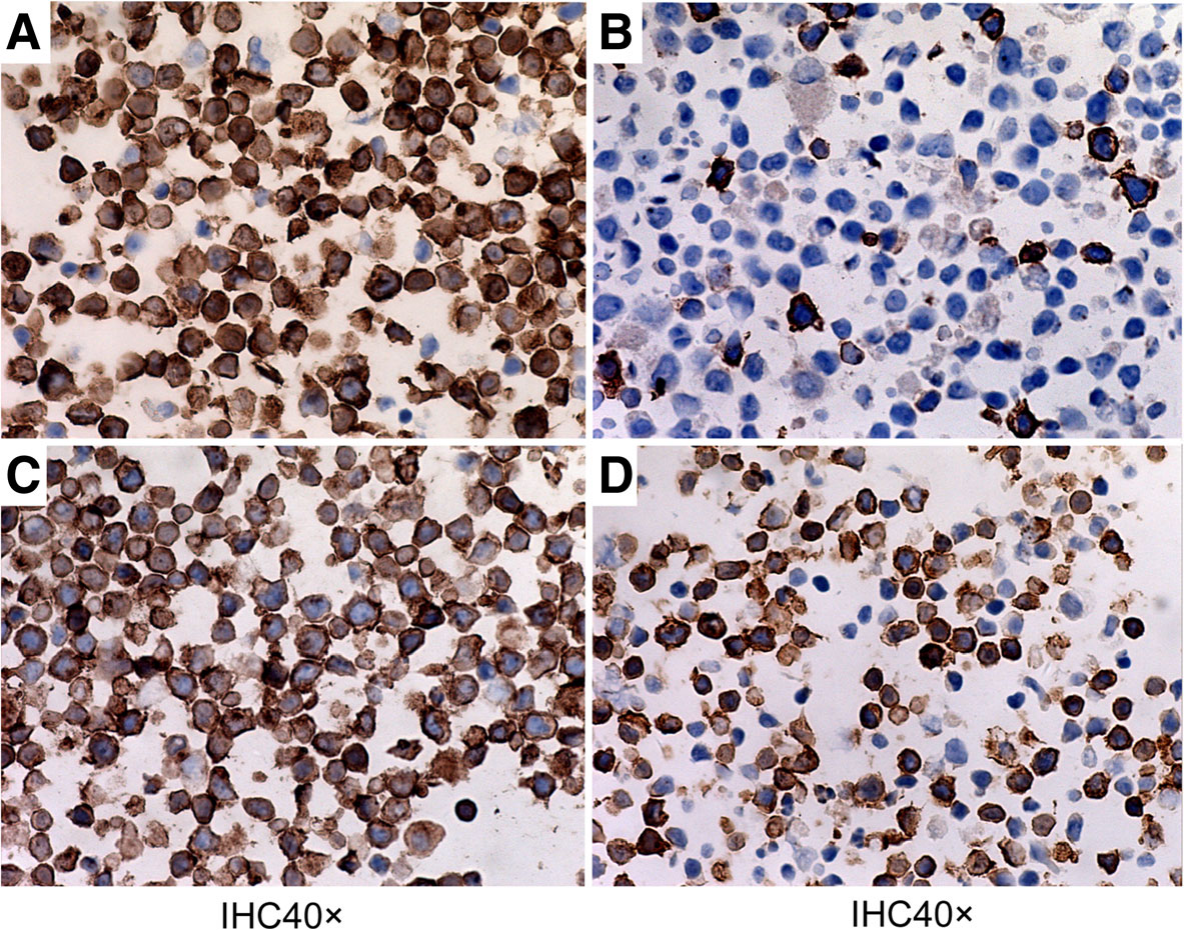
Immunohistochemical analysis of CIK cells. (**a**) The identification of CD3 on CIK cells (40×). (**b**) The identification of CD56 on CIK cells (40×). (**c**) The expression of CD46 on CIK cells (40×). (**d**) The efficiency of recombinant adenovirus infection of CIK cells (40×)

Immunohistochemical staining showed that prepared CIK cells expressed the CIK biomarkers CD3 and CD56 (Fig. 3a, b) as well as the KGHV500 receptor CD46 on the cell membrane (Fig. 3c). An Immunostain for the adenovirus hexon protein demonstrated that approximately 63.15% of CIK cells could bind with KGHV500 (Fig. 3d).

### The antitumor efficacy on tumor xenografts

**Fig. 4:**
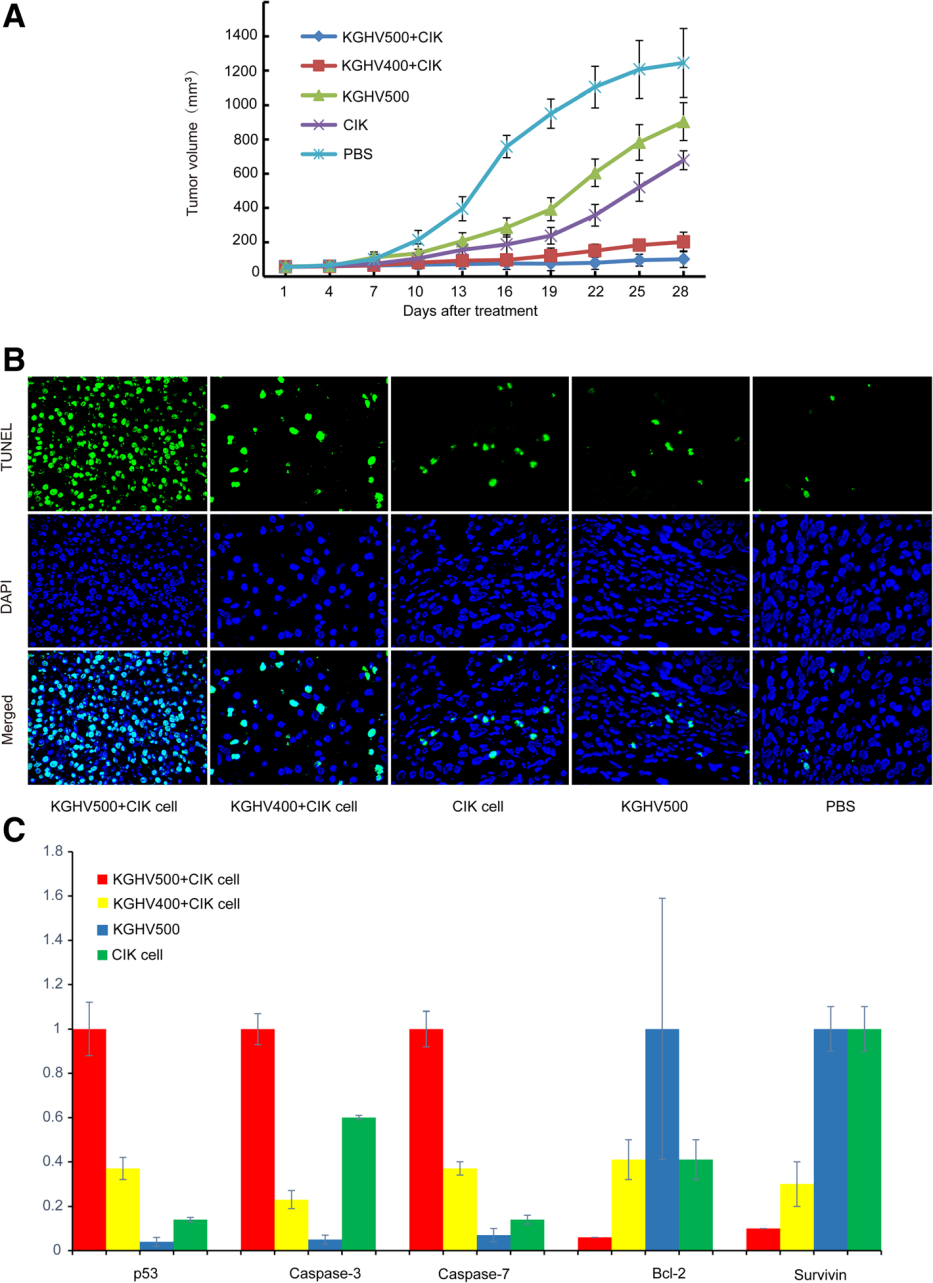
The antitumor efficacy against SW480 tumor xenografts. (**a**) Tumor size was measured every 3 d, and tumor volume was calculated. Tumor size decreased significantly in the KGHV500 combined with CIK treatment group. (**b**) There were more apoptotic tumor cells found in the KGHV500 combined with CIK cells group than in any other group (green: TUNEL, blue: DAPI × 1000). (**c**) The expression of caspase-3, caspase-7, p53, Bcl-2 and survivin genes in adenovirus or CIK cell-treated group detected by Q-PCR. p53, caspase-3 and caspase-7 gene expression was increased by KGHV500 combined with CIK cells treatment, and Bcl-2 and survivin gene expression was decreased by KGHV500 combined with CIK cells treatment

A nude mouse colorectal cancer xenograft model was used to investigate the antitumor efficacy of KGHV500. CIK cells carrying KGHV500, CIK cells carrying KGHV400, CIK cells, KGHV500 or PBS were injected into the caudal vein of the mice, and then were injected one time every other day. In comparison with the PBS group, we found that the average volumes (mm^3^) of tumors were reduced by 49% (CIK cells group), 84% (KGHV400 combined with CIK group), 27% (KGHV500 group) and 92% (KGHV500 combined with CIK cells group) on day 28 (*P* < 0.01) after initial virus injection (Fig. 4a). The tumor volumes in the combined KGHV500 and CIK treatment group was the smallest. These results demonstrated that KGHV500 combined with CIK treatment could significantly suppress tumor growth in vivo and achieve better antitumor effects than KGHV500, KGHV400 combined with CIK, CIK or PBS treatment alone.

We detected tumor cell apoptosis in xenografts with the TUNEL assay. The apoptosis rate exhibited by the KGHV500 combined with CIK cells treatment group 75 ± 16.68% was significantly higher than the rate in the KGHV400 combined with CIK cells treatment group 56 ± 10.52%, CIK cells treatment group 28 ± 7.65% or KGHV500 treatment group 23 ± 8.76% (*P* < 0.05). There was almost no apoptosis in the PBS treatment group 5 ± 3.36% (Fig. 4b). This implies that KGHV500 combined with CIK cells could significantly promote apoptosis in SW480 colorectal cancer cells and inhibit the growth of tumor xenografts in nude mice.

Furthermore, the apoptosis genes Bcl-2, caspase-3, caspase-7, p53 and survivin in xenografts were analyzed by Q-PCR, and the expression levels of the caspase-3, caspase-7, p53, Bcl-2 and survivin are shown in Fig. 4c. KGHV500 combined with CIK cells promoted the expression of caspase-3, caspase-7 and p53, and decreased the expression of Bcl-2 and survivin.

### KGHV500 in xenograft tumors and other organs

**Fig. 5:**
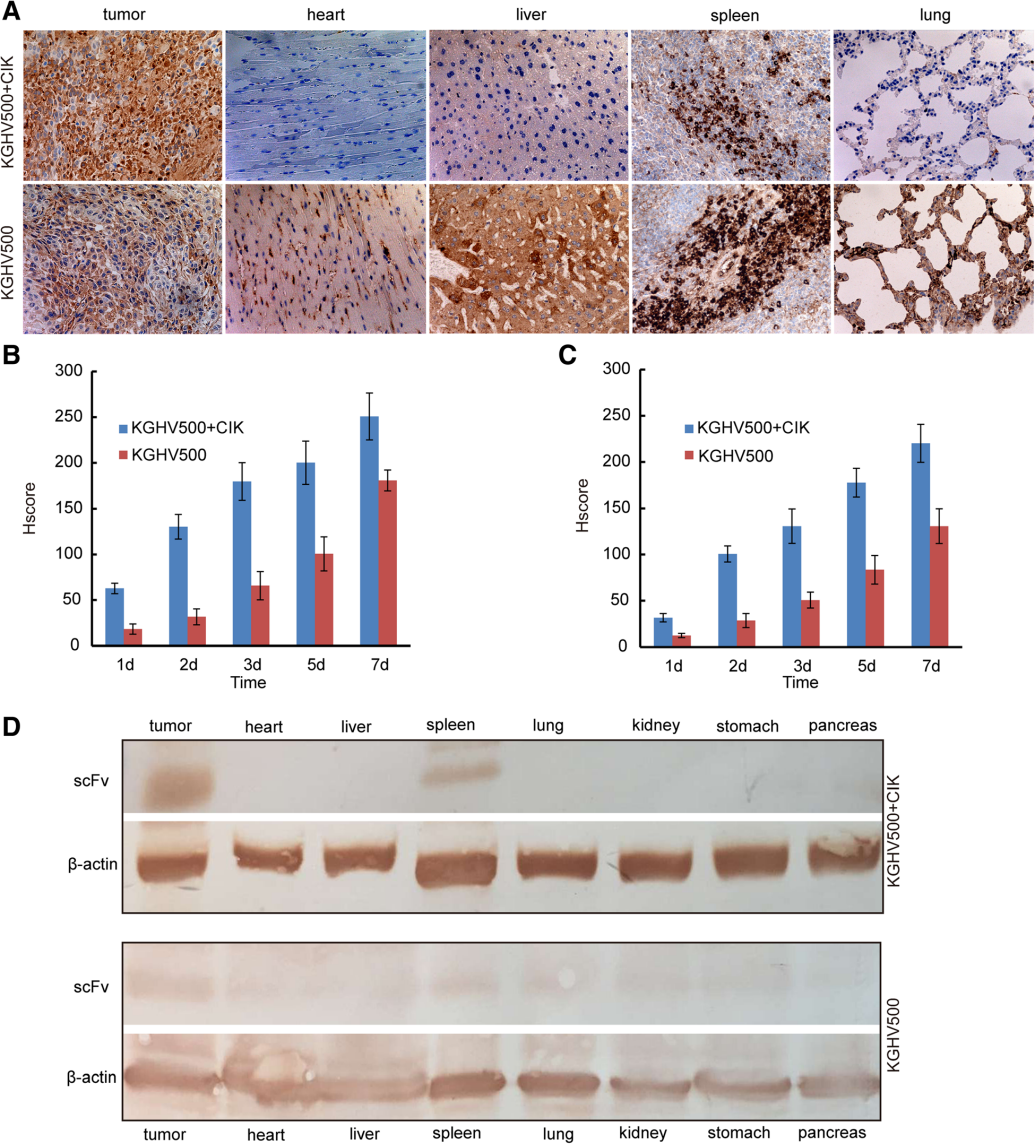
The expression of scFv and KGHV500 in xenograft tumors. (**a**) The expression of KGHV500 in xenograft tumor tissues (heart, liver, spleen and lung) was detected by immunohistochemistry after 7 days of treatment. There were more KGHV500 viruses in the KGHV500 combined with CIK cells treatment group. (**b**) The HSCORE of KGHV500, there were more positive cells in the KGHV500 combined with CIK cells group. (**c**) The HSCORE of scFv, there were more positive cells in the KGHV500 combined with CIK cells group. (**d**) The expression of scFv in organs (heart, liver, spleen, lung, kidney, stomach and pancreas) of nude mice treated with KGHV500 combined with CIK cells and KGHV500 was detected by Western blot. scFv was expressed only in the tumor and spleen in the KGHV500 combined with CIK cell treatment group, but expression of scFv was found in all tissues in the KGHV500 treatment group

A monoclonal antibody against the adenovirus hexon protein was used to detect KGHV500 in tumor tissues and mouse organs by immunohistochemistry. The HSCORE for the immunostaining is listed in Fig. 5b. In the KGHV500 combined with CIK treatment group there were many KGHV500 viruses in the tumor tissue but none in the heart, liver, or lung. Fewer KGHV500 viruses were observed in the tumor tissue from the KGHV500 treatment group compared with the KGHV500 combined with CIK treatment group (Fig. 5a). These results indicated that intravenous delivery of KGHV500 inside CIK cells was safe.

### The expression of scFv in xenograft tumors

Anti-p21Ras scFv was detected by immunohistochemistry. In the KGHV500 combined with CIK cells and the KGHV500 alone treatment groups, the HSCORE of scFv in the tumor tissue is shown in Fig. 5c. The results demonstrated that the expression of scFv in the KGHV500 combined with CIK cells group was higher than the expression in the KGHV500 group. Western blot analysis showed that scFv was expressed only in the tumor and spleen in the KGHV500 combined with CIK cells treatment group, and all other tissues were negative. However, in the KGHV500 treatment group, scFv expression was found in all tissues (Fig. 5d).

## Discussion

Gene therapy is a novel treatment approach in colorectal cancer, and its use is a developing trend in medicine. The occurrence of colorectal cancer is a multigene process involving the activation and inactivation of many oncogenes and tumor suppressor gene, respectively. In the current study, the tumor suppressor genes closely related to colorectal cancer were p53, APC and p16 [19–21]. To express the antitumor gene, intracellular antibodies are synthesized inside the cells from expression vectors, such as herpes simplex virus, adeno-associated virus, adenovirus, and retroviral vectors, that carry the antitumor gene [22]. Adenovirus vectors are constructed by deleting a region of the adenovirus, and inserting antibody genes to generate a recombinant adenovirus with the desired function [23]. These adenovirus vectors also have tissue or tissue-specific promoter sequences inserted into their genomes to ensure that expression of the antibody gene only inhibits tumors [24].

Breitbach et al. demonstrated that only a very small delivery of oncolytic vaccinia virus to tumors can be achieved in mouse experiment. [25] The adenovirus carrying the antitumor gene enters the body through intravenous injections, unfortunately, viral delivery is a critical problem in vivo. The most important thing is to guarantee that the adenoviral vector is successfully transported into the tumor where it expresses the antitumor gene.

Kwon et al. investigated the systemic delivery of oncolytic adenovirus via lipid, and the lipoplexes exhibited highly significant antitumor effects in vivo [26]. To reduce liver uptake and extend the lifespan in the blood, PEG has been conjugated to the surface of an adenovirus to make a “stealth” adenovirus [27], and PEGylated adenovirus exhibits increased plasma circulation and reduced liver toxicity [28]. Thorne et al. used CIK cells as vectors to carry poxvirus, and successfully prevented poxvirus escape from the immune system. The virus was accurately transported to the tumor by CIK cells [29]. CIK cells preinfected with viruses can efficiently maintain the ability to selectively target cancer cells and can be used as a delivery vehicle [6]. Thus, CIK cells could be used as a second vector.

To solve the problem of internal transport and enhance the antitumor effect of recombinant adenovirus KGHV500, in this study, CIK cells were used as a second vector to carry KGHV500 into the tumor. The migration and invasion abilities of SW480 cells were inhibited by KGHV500. Cell killing ability was measured by MTT assays, and KGHV500 significantly repressed the growth of SW480 cells. The significant antitumor activity of KGHV500 combined with CIK cells was observed in a nude mouse animal model with established colorectal tumors. In the KGHV500 combined with CIK cells treatment group, immunohistochemistry detected adenovirus expression in the tumor, a small amount of adenovirus expression in the spleen, which may be related to the homing of CIK cells to peripheral lymphoid organs [30], and no adenovirus in any other organs. A TUNEL assay detected that there were more apoptotic cells in tumors from the KGHV500 combined with CIK cells treatment group than in tumors from any other group. Anti-p21Ras scFv promoted the gene expression of caspase-3, caspase-7 and p53 (apoptosis-promoting genes) and decrease the expression of Bcl-2 and survivin genes.

## Conclusion

In summary, our experimental results confirmed that KGHV500 expressing anti-p21Ras scFv gene was successfully carried by CIK cells into tumors, and showing a significant inhibitory effect against the growth of CRC xenografts. Accordingly, the established therapeutic strategy of using KGHV500 combined with CIK is feasible and effective. This is very significant for clinical treatment for highly malignant CRC and other tumors with overexpressed p21Ras and Ras mutations. Our studies only used SW480 tumor cells and colorectal cancer nude mouse xenografts. In future studies, we hope to attempt to inhibit various primary tumors.

## Abbreviations

Ad: Adenovirus
CIK: Cytokine-induced killer
CRC: Colorectal cancer
EGFR: Epidermal growth factor receptor
FBS: Fetal bovine serum
HEK: Human embryonic kidney
HRP: Horseradish peroxidase
IHC: Immunohistochemistry
MTT: 3-(4,5-dimethyl-2-thiazolyl)-2,5-diphenyl-2-H-tetrazolium bromide
PVDF: Polyvinylidene fluoride
Q-PCR: Quantitative real-time PCR
scFv: Single chain fragment variable antibody
SPF: Specific pathogen free
